# Adherence to antidiabetic drug therapy and reduction of fatal events in elderly frail patients

**DOI:** 10.1186/s12933-023-01786-8

**Published:** 2023-03-10

**Authors:** Federico Rea, Laura Savaré, Valeria Valsassina, Stefano Ciardullo, Gianluca Perseghin, Giovanni Corrao, Giuseppe Mancia

**Affiliations:** 1 National Centre for Healthcare Research & Pharmacoepidemiology, at the University of Milano-Bicocca Milan, Milan, Italy; 2 Laboratory of Healthcare Research & Pharmacoepidemiology, Unit of Biostatistics, Epidemiology and Public Health, Department of Statistics and Quantitative Methods, University of Milano-Bicocca, Via Bicocca Degli Arcimboldi, 8, Edificio, U7, 20126 Milan, Italy; 3 MOX - Laboratory for Modeling and Scientific Computing, Department of Mathematics, Politecnico Di Milano, Milan, Italy; 4 CHDS - Center for Health Data Science, Human Technopole, Milan, Italy; 5 Department of Medicine and Rehabilitation, Policlinico Di Monza, Monza, Italy; 6 School of Medicine and Surgery, University of Milano Bicocca, Milan, Italy; 7 Directorate General for Health, Lombardy Region, Milan, Italy; 8 University of Milano-Bicocca (Emeritus Professor), Milan, Italy

**Keywords:** Frailty, Elderly, Antidiabetic drug therapy, Adherence, Mortality, Population-based study

## Abstract

**Background:**

To evaluate the protective effect of oral antidiabetic drugs in a large cohort of elderly patients with type 2 diabetes differing for age, clinical status, and life expectancy, including patients with multiple comorbidities and short survival.

**Methods:**

A nested case–control study was carried out by including the cohort of 188,983 patients from Lombardy (Italy), aged ≥ 65 years, who received ≥ 3 consecutive prescriptions of antidiabetic agents (mostly metformin and other older conventional agents) during 2012. Cases were the 49,201 patients who died for any cause during follow-up (up to 2018). A control was randomly selected for each case. Adherence to drug therapy was measured by considering the proportion of days of the follow-up covered by the drug prescriptions. Conditional logistic regression was used to model the risk of outcome associated with adherence to antidiabetic drugs. The analysis was stratified according to four categories of the clinical status (good, intermediate, poor, and very poor) differing for life expectancy.

**Results:**

There was a steep increase in comorbidities and a marked reduction of the 6-year survival from the very good to the very poor (or frail) clinical category. Progressive increase in adherence to treatment was associated with a progressive decrease in the risk of all-cause mortality in all clinical categories and at all ages (65–74, 75–84 and ≥ 85 years) except for the frail patient subgroup aged ≥ 85 years. The mortality reduction from lowest to highest adherence level showed a tendency to be lower in frail patients compared to the other categories. Similar although less consistent results were obtained for cardiovascular mortality.

**Conclusions:**

In elderly diabetic patients, increased adherence to antidiabetic drugs is associated with a reduction in the risk of mortality regardless of the patients’ clinical status and age, with the exception of very old patients (age ≥ 85 years) in the very poor or frail clinical category. However, in the frail patient category the benefit of treatment appears to be less than in patients in good clinical conditions.

**Supplementary Information:**

The online version contains supplementary material available at 10.1186/s12933-023-01786-8.

## Background

Randomized controlled trials have shown that, in patients with type 2 diabetes, old conventional antidiabetic drug therapy is associated with a reduction in the risk of microvascular complications [1] while meta-analyses of old conventional drug-based trials have shown that when data from several trials are pooled [2–6], the benefit of glucose-lowering treatment extends to macrovascular outcomes [7, 8]. Protection against macrovascular outcomes has been found to be even greater with use of newer antidiabetic agents such as sodium-glucose cotransporter 2 (SGLT2) inhibitors and glucagon-like peptide-1 receptor agonists (GLP1-RA), particularly for heart failure [9–12]. However, although randomized trial evidence on the protective effect of antidiabetic treatment has been extended to patients aged 65 years and beyond [13], knowledge is scanty for people aged 80 years or more [14] and especially limited for old patients with several comorbidities and a short life expectancy, i.e. those often referred to as “frail” patients [15], in part because frail patients tend to be excluded from recruitment in trials with a several year duration.

The objective of this observational study was to evaluate the protective effect of oral antidiabetic drugs in frail older adults (≥ 65 years) with type 2 diabetes. Frail individuals were identified via a multisource comorbidity score that accurately predicts the risk of mortality [16]. Analysis was extended to older adults in better clinical conditions.

## Methods

### Setting

The data used for the present study were retrieved from the healthcare utilization databases of Lombardy, a region of Italy that accounts for about 16% (almost 10 million people) of its population. All Italian citizens have equal access to healthcare services (e.g., hospitalizations, outpatient visits, instrumental examinations, laboratory tests, bioimaging, and drugs for chronic diseases) as part of the National Health Service (NHS). In Lombardy, these data are included in an automated system of databases that provides information on individual demographics, drug prescriptions (according to the Anatomical Therapeutic Chemical—ATC—system), medical or surgical interventions, and hospitalizations, according to diagnoses and procedures coded as in the International Classification of Diseases, Ninth Revision, Clinical Modification (ICD-9-CM) system. Because a unique identification code was used for all databases, their linkage provided information on the complete care pathway supplied to NHS beneficiaries for several years. To preserve privacy, in the analyses of the Lombardy databases each individual identification code is automatically deidentified, the inverse process being allowed only to the Regional Health Authority upon request from judicial authorities. A detailed description of the healthcare utilization databases of the Lombardy region in the field of cardiovascular and metabolic diseases is available in previous studies [17, 18]. The ICD-9-CM and ATC codes used for the current study are reported in Additional file 1: Table S1.

### Cohort selection

The target population consisted of the Lombardy residents aged ≥ 65 years who were NHS beneficiaries. Of these, those who received ≥ 3 consecutive prescriptions of oral antidiabetic agents during 2012 were identified and the date of the third prescription was defined as the index date. We considered that three consecutive prescriptions within a year are indicative of regular prescription and use. Because insulin might require frequent changes in dose requirements over time, patients prescribed only insulin were not included in the study. Two additional categories of patients were excluded from the analysis, i.e., those who had not been NHS beneficiaries for at least 5 years before the index date and those who did not reach at least 6 months of follow-up. The remaining patients were included into the final cohort whose members accumulated person-years of follow-up from the index date until the earliest date among death, emigration or June 30^th^, 2018.

### Selection of cases and controls

When the effect of time-dependent exposure needs to be investigated in the context of large databases, the nested case–control design is a valid alternative to the cohort design [19]. The case–control study consists of four steps: (i) cohort selection, (ii) case definition and selection, (iii) for each case, identification of all possible controls, and (iv) random selection of *m* controls for each case [20]. In the present study, the cohort involved the oral antidiabetic drug users as described above. Death from any cause was the primary outcome of interest, and cases were thus the cohort members who died during follow-up. For each case patient, all cohort members who survived when the matched case died were identified (i.e. the incidence density sampling method was adopted). For each case patient, one control was randomly selected from the cohort members to be matched for sex, age at index date, clinical status (see below) and date of index prescription.

A secondary outcome was cardiovascular mortality, i.e., death for ischemic heart disease, cerebrovascular disease, or heart failure, which was addressed by another nested case–control study in which patients who died for cardiovascular causes were the cases, and patients matched for age, sex and clinical status and index date were the controls, as described above.

### Assessing the clinical category

For each cohort member, the clinical status was assessed by the Multisource Comorbidity Score (MCS), i.e., a prognostic score based on 34 morbidities identified by the ICD-9-CM and ATC codes, which has been shown to predict mortality better than the Charlson, Elixahauser and Chronic Disease Scores in the Italian population [16, 21]. We assigned to each morbidity a weight proportional to its strength in predicting mortality, and calculated the sum of the morbidity' weights suffered by a patient. Because all cohort members suffered from diabetes, the contribution of diabetes to the MCS was not considered. Further details on the calculation of MCS are available in the original manuscript [16]. The score was used to separate patients according to 4 categories of clinical status: good (MCS = 0), intermediate (1 ≤ MCS ≤ 4), poor (5 ≤ MCS ≤ 14), and very poor (MCS ≥ 15).

### Adherence to oral antidiabetic drug treatment

For each patient, all antidiabetic drugs prescribed during the follow-up were identified. The period covered by an individual prescription was calculated by dividing the total amount of the drug prescribed for the defined daily dose. For overlapping prescriptions, the patient was assumed to have taken all drugs contained in the first prescription before starting the second one. Adherence was measured by the cumulative number of days in which the drug was available divided by the days of the follow-up, i.e. by the proportion of days covered (PDC) by treatment [22]. We classified patients prescribed more than one antidiabetic drug class as “adherent” if they were covered by at least one drug prescription. Because information on drug therapies dispensed during hospitalization was not available, the exposure to antidiabetic treatment before hospital admission was assumed to be continued for the entire span of the-hospital stay [23]. Four categories of adherence with antidiabetic drug therapy were considered, i.e. very low (≤ 25%), low (26%-50%), intermediate (51%-75%) and high (> 75%) PDC values. These cut-off values were used because in previous studies on the Lombardy database these adherence levels showed a clear association with mortality among elderly patients in treatment with antihypertensive and lipid-lowering drugs [24, 25].

### Covariates

Additional information included (i) the class of antidiabetic drugs, (ii) the use of insulin, (iii) comedications, e.g., use of antihypertensive, lipid-lowering, antiarrhythmic and other cardiovascular agents, and (iv) comorbidities, i.e., cardiovascular, kidney, respiratory disease, mental disorder, and cancer. Comedications and comorbidities were identified from out-of-hospital prescriptions and in-hospital diagnoses within the 5 years prior to the index date.

### Data analysis

Survival curves were built by means of the Kaplan–Meier method according with categories of clinical status, and compared through the log-rank test. Linear regression and chi square for the trend were used to test trend in covariates along the categories of clinical status. In addition, standardized mean differences were used to test differences between cases and controls. Standardized mean differences < 0.10 were considered negligible [26].

Conditional logistic regression models were fitted to estimate the odds ratio, and its 95% confidence interval (CI), of all-cause and cardiovascular mortality in relation to the PDC categories, using the lowest category (≤ 25%) as reference. Adjustments were made for the above-reported covariates. Odds ratio trends were tested according to the statistical significance of the regression coefficient of the recoded variable obtained by scoring the corresponding categories. All estimates were obtained by stratifying the cohort members according to the categories of clinical status. The impact of adherence on the outcomes according to categories of both clinical status and age (65–74 years, 75–84 years, and ≥ 85 years) was also measured.

### Sensitivity analysis

To verify the robustness of the main findings, five sensitivity analyses were performed. First, a different categorization of adherence was adopted: low (< 80%) and high (≥ 80%), as commonly used in the medical literature. Second, because the prescribed daily doses (not included in our database) might not be closely correspond to the defined daily doses [27], analyses were repeated by calculating the period covered by prescriptions from the number of tablets in the dispensed canisters, assuming a treatment schedule of one tablet per day. Third, the potential bias associated with unmeasured confounders was investigated by the rule-out approach described by Schneeweiss [28], which detects the extent of the unmeasured confounding required to fully account for the observed exposure–outcome association. We set the unmeasured confounder to exert a potentially marked effect on the results: (i) to have a 30% prevalence in the study population; (ii) to increase the risk of death up to tenfold in patients exposed to the unmeasured confounder than in those unexposed; and (iii) to be up to tenfold less common in high than in low adherent patients. Fourth, we investigated the possible presence of “healthy user bias”, i.e. the possibility that more adherent patients were more likely to follow healthy lifestyle advices or seek other preventive services than less adherent patients. The number of outpatient services (e.g. outpatient visits, laboratory examinations, instrument-based examinations) provided by the NHS in the previous 2 years was considered as a proxy for the patient’s behaviour to search for preventive non-pharmacological services. We proceeded with the following two-stage procedure: (i) ordinal logistic regression was fitted to estimate the odds ratio of adherence to antidiabetic treatment in relation to the number of outpatient services, and (ii) the association between adherence to antidiabetic treatment and survival was further investigated by stratifying for the number of outpatient services. Fifth, because the study cohort included prevalent users, i.e. patients already taking antidiabetic drug therapy before cohort entry, results might be affected by selection bias [29]. The analyses were then repeated by restricting the cohort to new users, i.e. patients who did not receive a prescription of antidiabetic drugs in the 5 years before the index date [30].

The Statistical Analysis System Software (version 9.4; SAS Institute, Cary, NC, USA) was used for the analyses. For all hypotheses tested, p-values less than 0.05 were considered to be significant.

### Data and resource availability

The data that support the findings of this study are available from Lombardy Region, but restrictions apply to the availability of these data, which were used under license for the current study, and so are not publicly available. Data are however available from the Lombardy Region upon reasonable request.

## Results

### Patients

**Fig. 1:**
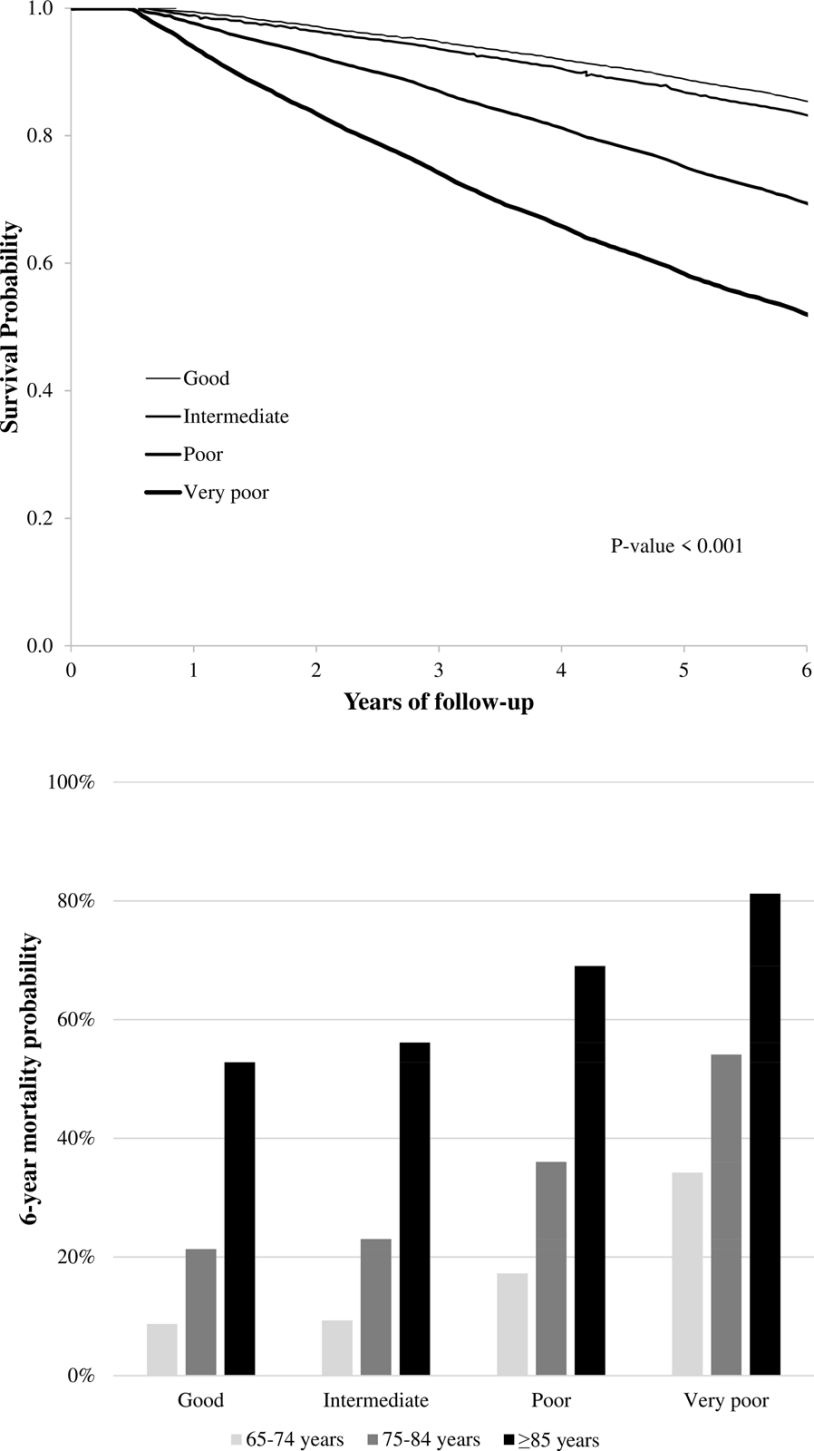
Kaplan–Meier survival curves for all-cause death according to the clinical category as determined by Multisource Comorbidity Score and 6-year mortality probabilities according to the clinical category and age strata

The distribution of exclusion criteria is reported in Additional file 1: Fig S1. Among the 276,336 patients on treatment with antidiabetic agents during 2012, 188,983 met the inclusion criteria. The cohort members accumulated 1,072,151 person-years of observation (on average, 5.7 years) and generated 49,219 deaths, with a mortality rate of 45.9 per 1000 person-years. As shown in Fig. 1 (upper panel), in the group as a whole the 6-year survival decreased from 85 to 52% from the group of patients with good to the group of patients with a very poor clinical status. Death incidence increased progressively as age increased and in each age group it was progressively greater as the clinical category changed from good to very poor or frail. More than 80% of patients aged ≥ 85 years with a very poor clinical status died during the study follow-up (Fig. 1, lower panel).

The baseline characteristics of cohort members are reported in Additional file 1: Table S2, according to the clinical category. Age and men prevalence increased as the clinical category deteriorated, this being the case also for use of cardiovascular drugs, non-cardiovascular drugs and previous hospitalization for a variety of diseases. The increase was particularly large for patients in the very poor clinical category. Among antidiabetic drugs, use of metformin and sulfonylurea were by far the most widely used drugs. Use of metformin, sulfonylurea, and pioglitazone decreased from the good to the very poor clinical category. The opposite trend was observed for the other antidiabetic drugs, including insulin and the much less frequently used newer oral antidiabetic agents.

Overall, 49,219 patients of the selected cohort died during follow-up (cases), of whom 49,201 were matched with alive patients from the cohort (controls). As expected, cases and controls had superimposable age, sex representation and clinical status (Table 1). This was the case also for the index date (see Methods). There were only small differences in virtually all clinical and therapeutic characteristics between the two groups. Compared to controls, cases showed an overall lower adherence to antidiabetic drug therapy.

**Table 1:** Comparison of demographic, clinical and therapeutic characteristics of the cohort members who died (cases) or survived (controls)

	Cases (n = 49,201)	Controls (n = 49,201)	SMD
Baseline			
Men	26,035 (52.9%)	26,035 (52.9%)	MV
Age (years): mean (SD)	79.7 (7.2)	79.6 (7.2)	MV
Clinical category^a^			MV
Good	6285 (12.8%)	6285 (12.8%)	
Intermediate	11,378 (23.1%)	11,378 (23.1%)	
Poor	22,717 (46.2%)	22,717 (46.2%)	
Very poor	8821 (17.9%)	8821 (17.9%)	
Antidiabetic agents at cohort entry			
Metformin	33,078 (67.2%)	34,031 (69.2%)	0.042
DPP-4 inhibitor	1869 (3.8%)	2348 (4.8%)	0.048
Sulfonylurea	25,767 (52.4)	25,127 (51.1%)	0.026
Pioglitazone	1991 (4.0%)	2316 (4.7%)	0.032
GLP-1 RA	293 (0.6%)	331 (0.7%)	0.010
Meglitinide	6917 (14.1%)	6064 (12.3%)	0.051
Alpha glucosidase inhibitors	1664 (3.4%)	1534 (3.1%)	0.015
Insulin	3675 (7.5%)	2919 (5.9%)	0.061
Other drugs			
Antihypertensive agents	45,601 (92.7%)	45,320 (92.1%)	0.022
Lipid-lowering agents	28,169 (57.3%)	30,063 (61.1%)	0.078
Antiarrhythmic agents	4835 (9.8%)	4312 (8.8%)	0.037
Antiplatelet drugs	33,169 (67.4%)	32,637 (66.3%)	0.023
Oral anticoagulant agents	7638 (15.5%)	6153 (12.5%)	0.087
Digitalis	4486 (9.1%)	3312 (6.7%)	0.088
Nitrates	11,453 (23.3%)	10,988 (22.3%)	0.023
Anti-gout drugs	10,466 (21.3%)	9674 (19.7%)	0.040
Antidepressant agents	12,294 (25.0%)	10,732 (21.8%)	0.075
Drugs for respiratory disease	17,010 (34.6%)	17,297 (35.2%)	0.012
Previous hospitalizations			
Cardiovascular disease	21,955 (44.6%)	20,014 (40.7%)	0.080
Kidney disease	2928 (6.0%)	2143 (4.4%)	0.072
Metal disorders	2159 (4.4%)	1658 (3.4%)	0.053
Respiratory disease	7347 (14.9%)	5240 (10.7%)	0.128
Cancer	7359 (15.0%)	7342 (14.9%)	0.001
During follow-up			
Adherence with antidiabetic drugs ^b^			0.213
Very low	4201 (8.5%)	3149 (6.4%)	
Low	10,272 (20.9%)	8400 (17.1%)	
Intermediate	13,437 (27.3%)	12,054 (24.5%)	
High	21,291 (43.3%)	25,598 (52.0%)	

*MV* matching variable, *SD* standard deviation, *SMD* standardized mean differences, *DDP-4* Dipeptidyl peptidase-4, *GLP-1 RA* glucagon-like peptide 1 receptor agonists

^a^ Clinical frailty was assessed by the Multisource Comorbidity Score (MCS) and four categories were considered: good (MCS = 0), intermediate (1 ≤ MCS ≤ 4), poor (5 ≥ MCS ≤ 14) and very poor (MCS ≥ 15)

^b^ Adherence was measured by the ratio between the days with available antidiabetic drug prescriptions and all days of follow up. Adherence categories are: very low: ≤ 25%; low: 26 to 50%; intermediate: 51 to 75%; and high: > 75%

### Antidiabetic drug therapy and mortality

**Fig. 2:**
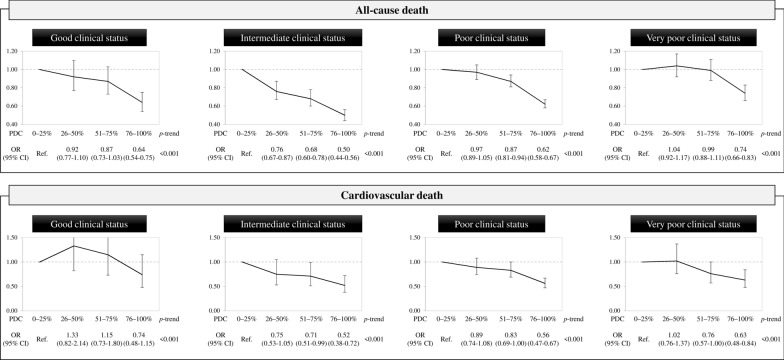
Effect of adherence with antidiabetic drugs on the odds ratio of all-cause and cardiovascular death according to the clinical category as measured by Multisource Comorbidity Score

The association between adherence to drug treatment and all-cause mortality is shown in Fig. 2, top panel. A progressive increase of adherence to treatment was associated with a progressive decrease in the risk of all-cause mortality in all clinical categories. The reduction of all-cause mortality from the lowest to the highest adherence level was lowest in the very poor clinical category compared to the other clinical categories, i.e. 26% (95% CI 17–34%) vs 36% (25–46%), 50% (44–56%) and 38% (33–42%) for the good, intermediate and poor clinical category, respectively. Similar trends were observed for cardiovascular mortality, i.e. the reduction from the lowest to the highest adherence level were 26% (-15–52%), 48% (28–62%), 44% (33–53%), and 37% (16–52%) for the good, intermediate and poor clinical category, respectively (Fig. 2, lower panels).

**Fig. 3:**
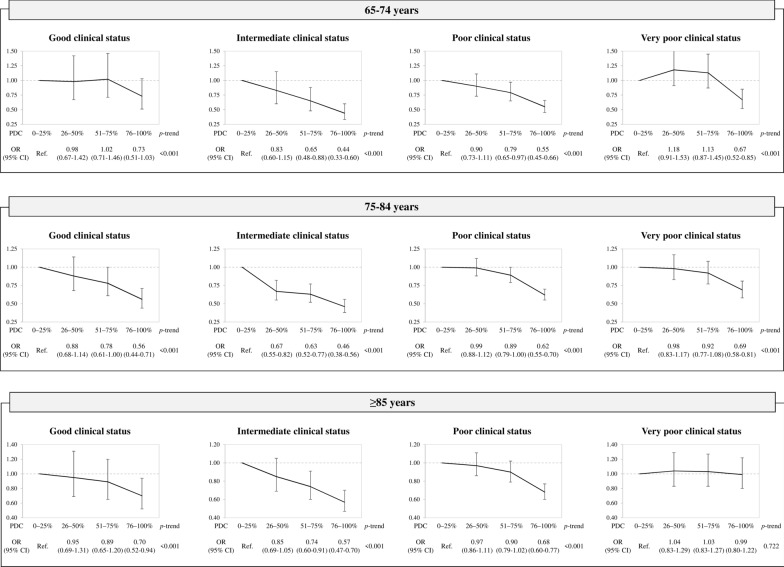
Effect of adherence with oral antidiabetics on the risk of all-cause mortality according to clinical category and age

The results of the stratified analysis for clinical category and age are reported in Fig. 3. In all age strata, there was an association between adherence to treatment and all-cause mortality, i.e. a decrease in the risk of fatal events from any cause as adherence increased. An exception was the group of patients aged ≥ 85 years with a very poor clinical status (or frailty) in whom changes in adherence did not modify the total mortality risk (p-trend = 0.722). The results for adherence and cardiovascular mortality were similar but less consistent (Additional file 1: Table S3). In the group of frail patients aged ≥ 85 years, an increase of adherence was associated with a 27% reduction of cardiovascular mortality, which did not achieve statistical significance (p-trend = 0.069). Furthermore, in the groups in good clinical conditions aged 65–74 years and aged ≥ 85 years, an increase of adherence had a paradoxical effect, i.e. an increase of cardiovascular mortality, albeit not significant (p-trend = 0.308 and 0.286, respectively). In these groups, however, the number of lethal cardiovascular events was extremely small (Additional file 1: Table S4).

### Sensitivity analyses

The results on all-cause mortality did not change either by varying the criteria for categorization of adherence with drug treatment (Additional file 1: Table S5) or by using a different way to estimate the duration of each prescription (Additional file 1: Table S6). As shown by the rule-out approach analysis reported in Additional file 1: Fig S2, assuming that highly adherent patients had a three-fold lower odds of exposure to an unmeasured confounder than patients with a very low adherence, the confounder should have increased the outcome risk of all-cause mortality by three-fold for nullifying the observed protective effect of drug adherence in patients with a very poor clinical category. The required nullifying confounder–outcome associations had to be even greater in the other clinical categories.

Additional file 1: Table S7 shows that the number of outpatient services used by the patients in the 2 years before the beginning of the study observations was associated with adherence to antidiabetic treatment. However, the results did not substantially change after accounting for this variable (Additional file 1: Table S8).

Finally, 61,722 cohort members were new users, i.e. they did not receive a prescription of antidiabetic drugs in the previous 5 years. Among these, 16,915 patients died during follow-up, of whom 16,897 were matched with alive new-user patients. The results did not change by restricting the cohort to new users (Additional file 1: Table S9).

## Discussion

Our study provides the following main findings. First, adherence to antidiabetic drug therapy reduced all-cause mortality in patients aged ≥ 65 years with type 2 diabetes. Second, this was the case regardless of the patients’ clinical status, i.e. antidiabetic treatment showed a protective effect not only in patients exhibiting relatively good clinical conditions but also in those with a progressive increase in the number of comorbidities, co-treatments, previous hospitalizations for a variety of diseases and a progressively marked reduction in the chance of survival, justifying their definition as “frail” individuals. Third, the reduction of all-cause mortality associated with better adherence to treatment was less pronounced in frail patients than in the other clinical categories. Furthermore, in frail patients there was no effect of antidiabetic treatment on survival from 85 years of age and beyond. These findings extend to the real-life setting the results of randomized clinical trials on the protective effect of antidiabetic drug treatment in patients aged ≥ 65 years [13]. They further suggest that protection extends to a very advanced age and that includes patients with a wide range of background clinical conditions and life expectancy, including those characterized by a high number of comorbidities, hospitalizations and risk of lethal events, which justifies their definition as frail patients. They also suggest, however, not only that in frail patients the protective effect of treatment on survival may be less than that seen in patients with better clinical conditions but fail to extend at or beyond 85 years of age.

Several other aspects of our study deserve to be mentioned. One, it is important to mention that “frail” patients aged 85 years or more were only 2644 out of 188,983 patients, i.e., about 1% of our cohort. Thus, only a very small fraction of old patients with diabetes may eventually fail to benefit from antidiabetic treatment and be candidates to a deprescribing decision [31]. Two, the results of our study that an increase of adherence to antidiabetic drug treatment was associated with a reduction of all-cause mortality in octogenarians patients expands available information which is scarce in people with diabetes in this age range. Three, the results obtained by the analysis of adherence to treatment and cardiovascular mortality exhibited trends that were in general similar to the trends exhibited by all-cause mortality, with, however, a lower level of consistency and some between clinical and adherence group differences that were not seen for all-cause mortality. We can speculate that a factor involved was the low number of cardiovascular lethal events, and thus the insufficient statistical power, in some groups, e.g. 4 and 22 lethal events in patients with very low adherence and an age of 65–74 and ≥ 85 years, respectively. Assuming that the proportion of highly adherent patients among controls is 89% (i.e. what was observed) and accepting a type 1 error of 0.05 with a statistical power of 80%, our study needed 1770 outcomes to detect a 25% significant reduction of mortality risk in people with high adherence to drug therapy. This number was not available in all subgroups and more frequently lower for cardiovascular than for all-cause mortality, making the latter a safer basis for conclusions. Four, the reduction of outcomes associated with higher levels of adherence to treatment may be originated by factors different from the increase of adherence to treatment, for example from the fact that patients adherent to drug treatment may also be also more prone to follow healthier lifestyles and control their health conditions via more frequent medical visits and laboratory or instrumental examinations. However, although the number of outpatient medical services utilized by more adherent patients was greater than that utilized by non-adherent ones, our findings provide evidence that the difference in the risk of mortality between low and high adherence to antidiabetic drugs did not disappear after accounting for this proxy measure of health seeking behaviour. This supports the conclusion that an increased adherence to antidiabetic drugs was responsible for the associated protective effect. Finally, albeit restricting cohort members to new users is considered one of the best approaches to reduce confounding in observational studies assessing the effectiveness of drug therapies [30], this reduces the generalizability of study results, especially among elderly frail patients. Indeed, only 33% of our cohort members did not have any prescription of antidiabetic drugs in the preceding years. Because the main results did not change by applying the new-user study design [30], this reinforces the robustness of the results.

Our study has several elements of strength as well as limitations. The strengths are that the study was based on a large and unselected population, which was made possible by the extension of the Italian healthcare system to virtually all citizens [32]. Furthermore, the drug prescription database we used provides accurate data because pharmacists are required to report prescriptions in detail in order to obtain reimbursement, and incorrect reports have legal consequences [33]. Finally, adoption of the “user-only” design (i.e. comparison between patients with the same indication at baseline, but with a different level of exposure to the drug of interest) reduces the potential for confounding [34]. Also, the choice of all-cause mortality as the primary outcome avoided any uncertainty about diagnostic accuracy in hospital records or causes of death reported in our database [35]. The limitations are that adherence to treatment was derived from drug prescriptions, a widely employed method to assess drug use in large populations which requires the assumption that the days covered by a prescription correspond to the days of drug use [32]. Because this is obviously not the case in all patients, our data on adherence to treatment are overestimated true adherence. Second, in absence of recorded daily doses of antidiabetic agents (not provided by our database [32]), we adopted the defined daily doses based on the reports of the World Health Organization (https://www.whocc.no/atc_ddd_index/) to estimate the time coverage of each prescription. However, the defined daily doses may overestimate the prescribed daily doses [27], making our adherence values lower than real adherence. However, it is unlikely that this had a substantial impact on the results because the main findings of the study were confirmed by a sensitivity analysis in which the drug coverage was estimated by the number of tablets in the dispensed canisters. Third, the inclusion and follow-up periods (from 2012 to 2018) did not allow us to suitably investigate the impact of the newer antidiabetic agents on mortality in different clinical categories and ages [9–12]. Between 2012 and 2018 treatment was still largely based on conventional antidiabetic drugs and only 3157 and 6771 cohort members started treatment with SGLT inhibitors and GLP1-RA, respectively.

Finally, and most importantly, because several clinical data (e.g. blood glucose glycated haemoglobin, lipid profile, blood pressure, body mass index, smoking, diet, duration of diabetes) are not included in the Lombardy database, we cannot exclude that a clinical imbalance between the adherence groups affected the results. However, our data were adjusted for several potential confounders. In addition, the “rule-out” sensitivity analysis showed that only a highly prevalent confounder closely associated with survival and extremely unbalanced between adherence groups could nullify the observed protection provided by greater adherence to antidiabetic drug therapy.

## Conclusions

In conclusion, adherence to antidiabetic drug therapy reduced the risk of death in elderly patients with diabetes, regardless of their background clinical conditions. The protective effect of treatment included patients definable as frail because of their high level of comorbidities, previous hospitalizations and short survival in whom in these patients the protective effect of antidiabetic treatment was less than that of patients in better clinical conditions but appreciable at least up to 85 years of age.

### Supplementary Information

**Additional file 1**: **Table S1**. Diagnostic and therapeutic codes used in the current study. **Table S2**. Comparison of demographic, clinical and therapeutic characteristics of the cohort members according to the clinical category. **Table S3**. Effect of adherence with oral antidiabetics on the risk of cardiovascular mortality according to categories of clinical frailty and age. **Table S4**. Number of cardiovascular deaths according to categories of clinical frailty and age. **Table S5**. Effect of adherence with antidiabetic drug therapy on the odds ratio (OR) of all-cause and cardiovascular death according to categorization of drug adherence by a different criterion than that used in the main analysis. **Table S6**. Effect of adherence with antidiabetic drug therapy on the odds ratio (OR) of all-cause death by assessing the coverage of each prescription from the number of tablets in the dispensed canister. **Table S7**. Effect of the number of outpatient services provided by the National Health Service on the odds ratio (OR) of adherence with antidiabetic drug therapy. **Table S8**. Effect of adherence with oral antidiabetics on the risk of all-cause mortality according to clinical categories of clinical frailty and number of outpatient services provided by the NHS in the previous two years. **Table S9**. Effect of adherence with oral antidiabetics on the risk of all-cause mortality by restricting the cohort to new users (i.e. patients with no antidiabetic drug prescriptions in the five years before the cohort entry). **Figure S1.** Flow-chart of inclusion and exclusion criteria for patients considered for data analysis.

## Data Availability

The data that support the findings of this study are available from Lombardy Region, but restrictions apply to the availability of these data, which were used under licence for the current study, and so are not publicly available. Data are however available from the Lombardy Region upon reasonable request.
