# Supplementary material for: Adherence to antidiabetic drug therapy and reduction of fatal events in elderly frail patients
Source: Cardiovasc Diabetol. 2023 Mar 10;22:53. doi: 10.1186/s12933-023-01786-8 (PMC9999593; doi:10.1186/s12933-023-01786-8)
Supplement: Supplementary file 1 — Additional file 1: Table S1. Diagnostic and therapeutic codes used in the current study. Table S2. Comparison of demographic, clinical and therapeutic characteristics of the cohort members according to the clinical category. Table S3. Effect of adherence with oral antidiabetics on the risk of cardiovascular mortality according to categories of clinical frailty and age. Table S4. Number of cardiovascular deaths according to categories of clinical frailty and age. Table S5. Effect of adherence with antidiabetic drug therapy on the odds ratio (OR) of all-cause and cardiovascular death according to categorization of drug adherence by a different criterion than that used in the main analysis. Table S6. Effect of adherence with antidiabetic drug therapy on the odds ratio (OR) of all-cause death by assessing the coverage of each prescription from the number of tablets in the dispensed canister. Table S7. Effect of the number of outpatient services provided by the National Health Service on the odds ratio (OR) of adherence with antidiabetic drug therapy. Table S8. Effect of adherence with oral antidiabetics on the risk of all-cause mortality according to clinical categories of clinical frailty and number of outpatient services provided by the NHS in the previous two years. Table S9. Effect of adherence with oral antidiabetics on the risk of all-cause mortality by restricting the cohort to new users (i.e. patients with no antidiabetic drug prescriptions in the five years before the cohort entry). Figure S1. Flow-chart of inclusion and exclusion criteria for patients considered for data analysis. [file 12933_2023_1786_MOESM1_ESM.docx]

**Adherence to antidiabetic drug therapy and reduction of fatal events in elderly frail patients**

Federico REA ^1,2^, Laura SAVARé ^1,3,4^, Valeria VALSASSINA ^2^, Stefano CIARDULLO ^5,6^, Gianluca PERSEGHIN ^5,6^, Giovanni CORRAO ^1,2,7^, Giuseppe MANCIA ^8^

^1^ National Centre for Healthcare Research & Pharmacoepidemiology, at the University of Milano-Bicocca Milan, Italy

^2^ Laboratory of Healthcare Research & Pharmacoepidemiology, Unit of Biostatistics, Epidemiology and Public Health, Department of Statistics and Quantitative Methods, University of Milano-Bicocca, Milan, Italy

^3^ MOX - Laboratory for Modeling and Scientific Computing, Department of Mathematics, Politecnico di Milano, Milan, Italy

^4^ CHDS - Center for Health data Science, Human Technopole, Milan, Italy

^5^ Department of Medicine and Rehabilitation, Policlinico di Monza, Monza, Italy

^6^ School of Medicine and Surgery, University of Milano Bicocca, Milan, Italy

^7^ Directorate General for Health, Lombardy Region, Milan, Italy

^8^ University of Milano-Bicocca (Emeritus Professor), Milan, Italy

**SUPPLEMENTARY MATERIAL**

**Supplementary Table S1**. Diagnostic and therapeutic codes used in the current study

|  | **Codes** |
| --- | --- |
| **Diseases (hospitalization)** † |  |
| Cardiovascular events | 390 – 459.x |
| Diabetes | 250.x |
| Kidney disease | 584.x – 586 |
| Respiratory disease | 460 – 519.x |
| Mental disorders | 290.x – 319 |
| Cancer | 140.x – 239.x |
| **Cause of death** ξ |  |
| Ischemic heart disease | I20 – I25 |
| Cerebrovascular disease | I60 – I69 |
| Heart failure | I50 |
| **Drugs** § |  |
| Antidiabetic drugs | A10 |
| Metformin | A10BA02, A10BD02, A10BD05, A10BD07, A10BD08 |
| DPP-4 inhibitor | A10BH, A10BD07, A10BD08 |
| Sulfonylurea | A10BB, A10BD01, A10BD02, A10BD06 |
| Pioglitazone | A10BG03, A10BD05, A10BD06 |
| GLP-1 RA | A10BJ |
| Meglitinide | A10BX |
| Alpha glucosidase inhibitors | A10BF |
| Insulin | A10A |
| Lipid-lowering drugs | C10 |
| Antihypertensive agents | C02, C03, C07, C08, C09 |
| Antiarrhythmic agents | C01B |
| Antiplatelet drugs | B01AC |
| Oral anticoagulant agents | B01AA, B01AE, B01AF |
| Anti-gout | M04 |
| Digitalis | C01AA |
| Nitrates | C01DA |
| NSAIDs | M01A |
| Antidepressant agents | N06A |
| Drugs for respiratory disease | R03 |

† According to the ICD-9-CM (International Classification of Disease, 9th Revision) system

ξ According to the ICD-10-CM (International Classification of Disease, 10th Revision) system

§ According to the ATC (Anatomical-Therapeutic-Chemical) classification system

**Supplementary Table S2**. Comparison of demographic, clinical and therapeutic characteristics of the cohort members according to the clinical category

|  | Clinical category ^*^ | | | | p-value |
| --- | --- | --- | --- | --- | --- |
|  | Good  (n=39,919) | Intermediate  (n=62,531) | Poor  (n=69,042) | Very poor ^†^  (n=17,491) |  |
| **Baseline** |  |  |  |  |  |
| Men | 21,675 (54.3%) | 30,792 (49.2%) | 34,305 (49.7%) | 9,531 (54.5%) | <0.001 |
| Age (years): mean (SD) | 73.69 (6.33) | 74.53 (6.51) | 76.61 (7.03) | 76.96 (6.85) | <0.001 |
| Antidiabetic agents at cohort entry |  |  |  |  |  |
| Metformin | 31,055 (77.8%) | 48,144 (77.0%) | 50,287 (72.8%) | 11,410 (65.2%) | <0.001 |
| DPP-4 inhibitor | 2,464 (6.2%) | 3,933 (6.3%) | 3,960 (5.7%) | 875 (5.0%) | <0.001 |
| Sulfonylurea | 20,902 (52.4%) | 30,919 (49.4%) | 32,666 (47.3%) | 7,310 (41.8%) | <0.001 |
| Pioglitazone | 3,076 (7.7%) | 4,360 (7.0%) | 3,441 (5.0%) | 613 (3.5%) | <0.001 |
| GLP-1 RA | 334 (0.8%) | 689 (1.1%) | 806 (1.2%) | 165 (0.9%) | <0.001 |
| Meglitinide | 2,165 (5.4%) | 4,234 (6.8%) | 8,044 (11.7%) | 3,355 (19.2%) | <0.001 |
| Alpha glucosidase inhibitors | 980 (2.5%) | 1,614 (2.6%) | 2,109 (3.1%) | 724 (4.1%) | <0.001 |
| Insulin | 1,546 (3.9%) | 3,168 (5.1%) | 5,313 (7.7%) | 1,637 (9.4%) | <0.001 |
| Other drugs |  |  |  |  |  |
| Antihypertensive agents | 30,878 (77.4%) | 54,237 (86.7%) | 65,807 (95.3%) | 16,695 (95.4%) | <0.001 |
| Lipid-lowering agents | 21,938 (55.0%) | 38,704 (61.9%) | 47,383 (68.6%) | 11,463 (65.5%) | <0.001 |
| Antiarrhythmic agents | 2 (0.0%) | 2,537 (3.8%) | 7,602 (11.0%) | 2,581 (14.8%) | <0.001 |
| Antiplatelet drugs | 15,398 (38.6%) | 34,410 (55.0%) | 49,905 (72.3%) | 12,833 (73.4%) | <0.001 |
| Oral anticoagulant agents | 0 (0.0%) | 2,575 (4.1%) | 11,341 (16.4%) | 3,558 (20.3%) | <0.001 |
| Digitalis | 0 (0.0%) | 325 (0.5%) | 6,099 (8.8%) | 1,852 (10.6%) | <0.001 |
| Nitrates | 0 (0.0%) | 2,648 (4.2%) | 20,762 (30.1%) | 5,649 (32.3%) | <0.001 |
| Anti-gout drugs | 14 (0.0%) | 7,059 (11.3%) | 15,765 (22.8%) | 5,845 (33.4%) | <0.001 |
| Antidepressant agents | 4,151 (10.4%) | 10,185 (16.3%) | 16,675 (24.2%) | 5,904 (33.8%) | <0.001 |
| Drugs for respiratory disease | 1,810 (4.5%) | 19,968 (31.9%) | 28,126 (40.7%) | 8,411 (48.1%) | <0.001 |
| Previous hospitalizations |  |  |  |  |  |
| Cardiovascular disease | 1,530 (3.8%) | 11,219 (17.9%) | 35,046 (50.8%) | 12,310 (70.4%) | <0.001 |
| Kidney disease | 29 (0.1%) | 180 (0.3%) | 2,176 (3.2%) | 2,710 (15.5%) | <0.001 |
| Mental disorders | 83 (0.2%) | 391 (0.6%) | 2,129 (3.1%) | 1,192 (11.4%) | <0.001 |
| Respiratory disease | 381 (1.0%) | 2,035 (3.8%) | 8,248 (11.9%) | 4,635 (26.5%) | <0.001 |
| Cancer | 976 (2.4%) | 2,372 (3.8%) | 7,489 (10.8%) | 9,258 (52.9%) | <0.001 |

SD: standard deviation; DDP-4: Dipeptidyl peptidase-4; GLP-1 RA: glucagon-like peptide 1 receptor agonists

^*^ Clinical status was assessed by the Multisource Comorbidity Score (MCS) and four categories were considered: good (MCS=0), intermediate (1 ≤ MCS ≤ 4), poor (5 ≤ MCS ≤ 14) and very poor (MCS≥15).

^†^ The very poor clinical status was regarded as representative of frail patients

**Supplementary Table S3**. Effect of adherence with oral antidiabetics on the risk of cardiovascular mortality according to categories of clinical frailty and age

|  |  | Clinical frailty ^*^ | | | |
| --- | --- | --- | --- | --- | --- |
| **Age strata** | Adherence | Good | Intermediate | Poor | Very poor |
| 65-74 | Very low | 1.00 (Ref.) | 1.00 (Ref.) | 1.00 (Ref.) | 1.00 (Ref.) |
|  | Low | 3.25 (0.67 to 15.81) | 1.89 (0.57 to 6.32) | 1.02 (0.55 to 1.89) | 1.13 (0.45 to 2.84) |
|  | Intermediate | 3.10 (0.70 to 14.33) | 0.86 (0.26 to 2.78) | 0.83 (0.46 to 1.49) | 0.59 (0.23 to 1.48) |
|  | High | 2.19 (0.49 to 9.77) | 0.65 (0.21 to 2.04) | 0.56 (0.32 to 0.99) | 0.31 (0.12 to 0.79) |
|  | p-trend | 0.308 | <0.001 | <0.001 | <0.001 |
| 75-84 | Very low | 1.00 (Ref.) | 1.00 (Ref.) | 1.00 (Ref.) | 1.00 (Ref.) |
|  | Low | 0.78 (0.36 to 1.71) | 0.62 (0.37 to 1.03) | 0.87 (0.66 to 1.15) | 0.92 (0.59 to 1.42) |
|  | Intermediate | 0.79 (0.39 to 1.59) | 0.60 (0.37 to 0.97) | 0.76 (0.58 to 0.99) | 0.74 (0.49 to 1.13) |
|  | High | 0.39 (0.19 to 0.80) | 0.47 (0.30 to 0.74) | 0.52 (0.40 to 0.68) | 0.66 (0.44 to 1.00) |
|  | p-trend | <0.001 | <0.001 | <0.001 | 0.014 |
| ≥85 | Very low | 1.00 (Ref.) | 1.00 (Ref.) | 1.00 (Ref.) | 1.00 (Ref.) |
|  | Low | 2.04 (0.92 to 4.53) | 0.76 (0.45 to 1.29) | 0.90 (0.68 to 1.19) | 1.00 (0.62 to 1.60) |
|  | Intermediate | 1.51 (0.69 to 3.30) | 0.92 (0.56 to 1.50) | 0.95 (0.72 to 1.25) | 0.73 (0.46 to 1.14) |
|  | High | 1.14 (0.56 to 2.31) | 0.61 (0.37 to 1.01) | 0.62 (0.48 to 0.81) | 0.73 (0.47 to 1.13) |
|  | p-trend | 0.286 | 0.042 | <0.001 | 0.069 |

^*^ Clinical frailty was assessed by the Multisource Comorbidity Score (MCS) and four categories were considered: good (MCS=0), intermediate (MCS ≥ 1 to ≤ 4), poor (MCS ≥ 5 to ≤ 14) and very poor (MCS≥15).

ORs (and 95% confidence intervals, CI) was estimated with conditional logistic regression. Estimates were adjusted for the covariates listed in Table 1

Adherence to treatment was measured by the ratio between the days with available antidiabetic drug prescriptions and all days of follow up. Adherence categories are: very low: ≤25%; low: 26 to 50%; intermediate: 51 to 75%; and high: >75%.

**Supplementary Table S4**. Number of cardiovascular deaths according to categories of clinical frailty and age

|  |  | **Clinical category** | | | |
| --- | --- | --- | --- | --- | --- |
| **Age strata** | Adherence | Good | Intermediate | Poor | Very poor |
| 65-74 | Very low | 4 | 11 | 51 | 26 |
|  | Low | 37 | 70 | 148 | 71 |
|  | Intermediate | 66 | 82 | 214 | 70 |
|  | High | 141 | 213 | 423 | 103 |
| 75-84 | Very low | 25 | 65 | 194 | 82 |
|  | Low | 69 | 139 | 442 | 189 |
|  | Intermediate | 135 | 222 | 600 | 215 |
|  | High | 177 | 453 | 889 | 295 |
| ≥85 | Very low | 22 | 54 | 183 | 72 |
|  | Low | 63 | 133 | 392 | 131 |
|  | Intermediate | 66 | 179 | 493 | 142 |
|  | High | 98 | 213 | 643 | 182 |

**Supplementary Table S5**. Effect of adherence with antidiabetic drug therapy on the odds ratio (OR) of all-cause and cardiovascular death according to categorization of drug adherence by a different criterion than that used in the main analysis.

| Clinical status ^a^ | Adherence with antidiabetic drug therapy | All-cause mortality | Cardiovascular mortality |
| --- | --- | --- | --- |
|  |  | OR (95% CI) | OR (95% CI) |
| Good | PDC < 80% | 1.00 (Reference) | 1.00 (Reference) |
|  | PDC ≥ 80% | 0.69 (0.64 to 0.75) | 0.60 (0.49 to 0.73) |
| Intermediate | PDC < 80% | 1.00 (Reference) | 1.00 (Reference) |
|  | PDC ≥ 80% | 0.65 (0.62 to 0.69) | 0.68 (0.59 to 0.79) |
| Poor | PDC < 80% | 1.00 (Reference) | 1.00 (Reference) |
|  | PDC ≥ 80% | 0.66 (0.64 to 0.69) | 0.63 (0.57 to 0.69) |
| Very poor | PDC < 80% | 1.00 (Reference) | 1.00 (Reference) |
|  | PDC ≥ 80% | 0.71 (0.66 to 0.77) | 0.69 (0.58 to 0.82) |

PDC: Proportion of Days Covered

^a^ Clinical status was assessed by the Multisource Comorbidity Score and four categories were considered: good (score=0), intermediate (1 ≤ score ≤ 4), poor (5 ≤ score ≤ 14) and very poor (score≥15).

**Supplementary Table S6**. Effect of adherence with antidiabetic drug therapy on the odds ratio (OR) of all-cause death by assessing the coverage of each prescription from the number of tablets in the dispensed canister

| Clinical status ^a^ | Adherence with antidiabetic drug therapy | OR | 95% CI |
| --- | --- | --- | --- |
| Good | PDC ≤ 25% | 1.00 | Reference |
|  | 26% ≤ PDC ≤ 50% | 1.11 | 0.84 to 1.48 |
|  | 51% ≤ PDC ≤ 75% | 1.05 | 0.80 to 1.36 |
|  | PDC > 75% | 0.56 | 0.44 to 0.71 |
|  | p-trend | <0.001 | |
| Intermediate | PDC ≤ 25% | 1.00 | Reference |
|  | 26% ≤ PDC ≤ 50% | 1.01 | 0.82 to 1.24 |
|  | 51% ≤ PDC ≤ 75% | 0.85 | 0.70 to 1.02 |
|  | PDC > 75% | 0.48 | 0.40 to 0.57 |
|  | p-trend | <0.001 | |
| Poor | PDC ≤ 25% | 1.00 | Reference |
|  | 26% ≤ PDC ≤ 50% | 1.13 | 1.00 to 1.28 |
|  | 51% ≤ PDC ≤ 75% | 1.09 | 0.97 to 1.22 |
|  | PDC > 75% | 0.65 | 0.58 to 0.72 |
|  | p-trend | <0.001 | |
| Very poor | PDC ≤ 25% | 1.00 | Reference |
|  | 26% ≤ PDC ≤ 50% | 1.16 | 0.97 to 1.40 |
|  | 51% ≤ PDC ≤ 75% | 1.34 | 1.13 to 1.60 |
|  | PDC > 75% | 0.80 | 0.69 to 0.94 |
|  | p-trend | <0.001 | |

PDC: Proportion of Days Covered

^a^ Clinical status was assessed by the Multisource Comorbidity Score and four categories were considered: good (score=0), intermediate (1 ≤ score ≤ 4), poor (5 ≤ score ≤ 14) and very poor (score≥15).

**Supplementary Table S7**. Effect of the number of outpatient services provided by the National Health Service on the odds ratio (OR) of adherence with antidiabetic drug therapy

| Number of outpatient services | OR | 95% CI |
| --- | --- | --- |
| <50 | 1.00 | Ref. |
| 50-150 | 1.07 | 1.03 to 1.11 |
| >150 | 1.13 | 1.08 to 1.17 |
| p-trend | <0.001 | |

Estimates were adjusted for the covariates listed in Table 1.

**Supplementary Table S8**. Effect of adherence with oral antidiabetics on the risk of all-cause mortality according to clinical categories of clinical frailty and number of outpatient services provided by the NHS in the previous two years

| Number of outpatient services |  | Clinical category ^a^ | | | |
| --- | --- | --- | --- | --- | --- |
|  | Adherence | Good | Intermediate | Poor | Very poor ^b^ |
| <50 | Very low | 1.00 (Ref.) | 1.00 (Ref.) | 1.00 (Ref.) | 1.00 (Ref.) |
|  | Low | 0.82 (0.62 to 1.08) | 0.87 (0.69 to 1.10) | 0.87 (0.72 to 1.04) | 1.18 (0.78 to 1.79) |
|  | Intermediate | 0.76 (0.58 to 0.98) | 0.72 (0.58 to 0.91) | 0.76 (0.63 to 0.90) | 0.78 (0.52 to 1.18) |
|  | High | 0.54 (0.42 to 0.69) | 0.55 (0.45 to 0.69) | 0.51 (0.43 to 0.61) | 0.66 (0.44 to 0.98) |
|  | p-trend | <0.001 | <0.001 | <0.001 | 0.001 |
| 50-150 | Very low | 1.00 (Ref.) | 1.00 (Ref.) | 1.00 (Ref.) | 1.00 (Ref.) |
|  | Low | 1.09 (0.84 to 1.41) | 0.75 (0.62 to 0.90) | 1.01 (0.90 to 1.13) | 1.06 (0.88 to 1.28) |
|  | Intermediate | 1.05 (0.81 to 1.34) | 0.68 (0.56 to 0.81) | 0.91 (0.81 to 1.01) | 1.08 (0.91 to 1.29) |
|  | High | 0.77 (0.60 to 0.98) | 0.49 (0.41 to 0.58) | 0.67 (0.60 to 0.75) | 0.79 (0.66 to 0.94) |
|  | p-trend | <0.001 | <0.001 | <0.001 | <0.001 |
| >150 | Very low | 1.00 (Ref.) | 1.00 (Ref.) | 1.00 (Ref.) | 1.00 (Ref.) |
|  | Low | 0.61 (0.34 to 1.08) | 0.64 (0.45 to 0.92) | 1.02 (0.87 to 1.20) | 0.97 (0.81 to 1.16) |
|  | Intermediate | 0.62 (0.35 to 1.10) | 0.59 (0.42 to 0.84) | 0.95 (0.81 to 1.10) | 0.92 (0.77 to 1.09) |
|  | High | 0.59 (0.33 to 1.03) | 0.43 (0.31 to 0.61) | 0.65 (0.56 to 0.76) | 0.70 (0.59 to 0.83) |
|  | p-trend | 0.188 | <0.001 | <0.001 | <0.001 |

^a^ Clinical status was assessed by the Multisource Comorbidity Score and four categories were considered: good (score=0), intermediate (1 ≤ score ≤ 4), poor (5 ≤ score ≤ 14) and very poor (score≥15)

^b^ The very poor clinical category was regarded as representative of frail patients

**Supplementary Table S9**. Effect of adherence with oral antidiabetics on the risk of all-cause mortality by restricting the cohort to new users (i.e. patients with no antidiabetic drug prescriptions in the five years before the cohort entry)

| Clinical status ^a^ | Adherence with antidiabetic drug therapy | OR | 95% CI |
| --- | --- | --- | --- |
| Good | PDC ≤ 25% | 1.00 | Reference |
| (n=4,340) | 26% ≤ PDC ≤ 50% | 0.83 | 0.59 to 1.18 |
|  | 51% ≤ PDC ≤ 75% | 0.74 | 0.54 to 1.03 |
|  | PDC > 75% | 0.46 | 0.33 to 0.63 |
|  | p-trend | <0.001 | |
| Intermediate | PDC ≤ 25% | 1.00 | Reference |
| (n=7,888) | 26% ≤ PDC ≤ 50% | 0.87 | 0.69 to 1.11 |
|  | 51% ≤ PDC ≤ 75% | 0.67 | 0.54 to 0.85 |
|  | PDC > 75% | 0.47 | 0.38 to 0.58 |
|  | p-trend | <0.001 | |
| Poor | PDC ≤ 25% | 1.00 | Reference |
| (n=15,692) | 26% ≤ PDC ≤ 50% | 0.94 | 0.81 to 1.09 |
|  | 51% ≤ PDC ≤ 75% | 0.82 | 0.71 to 0.94 |
|  | PDC > 75% | 0.55 | 0.48 to 0.63 |
|  | p-trend | <0.001 | |
| Very poor | PDC ≤ 25% | 1.00 | Reference |
| (n=5,874) | 26% ≤ PDC ≤ 50% | 1.14 | 0.91 to 1.42 |
|  | 51% ≤ PDC ≤ 75% | 0.99 | 0.80 to 1.23 |
|  | PDC > 75% | 0.71 | 0.58 to 0.87 |
|  | p-trend | <0.001 | |

PDC: Proportion of Days Covered

^a^ Clinical status was assessed by the Multisource Comorbidity Score and four categories were considered: good (score=0), intermediate (1 ≤ score ≤ 4), poor (5 ≤ score ≤ 14) and very poor (score≥15).

**Supplementary Figure S1.** Flow-chart of inclusion and exclusion criteria for patients considered for data analysis


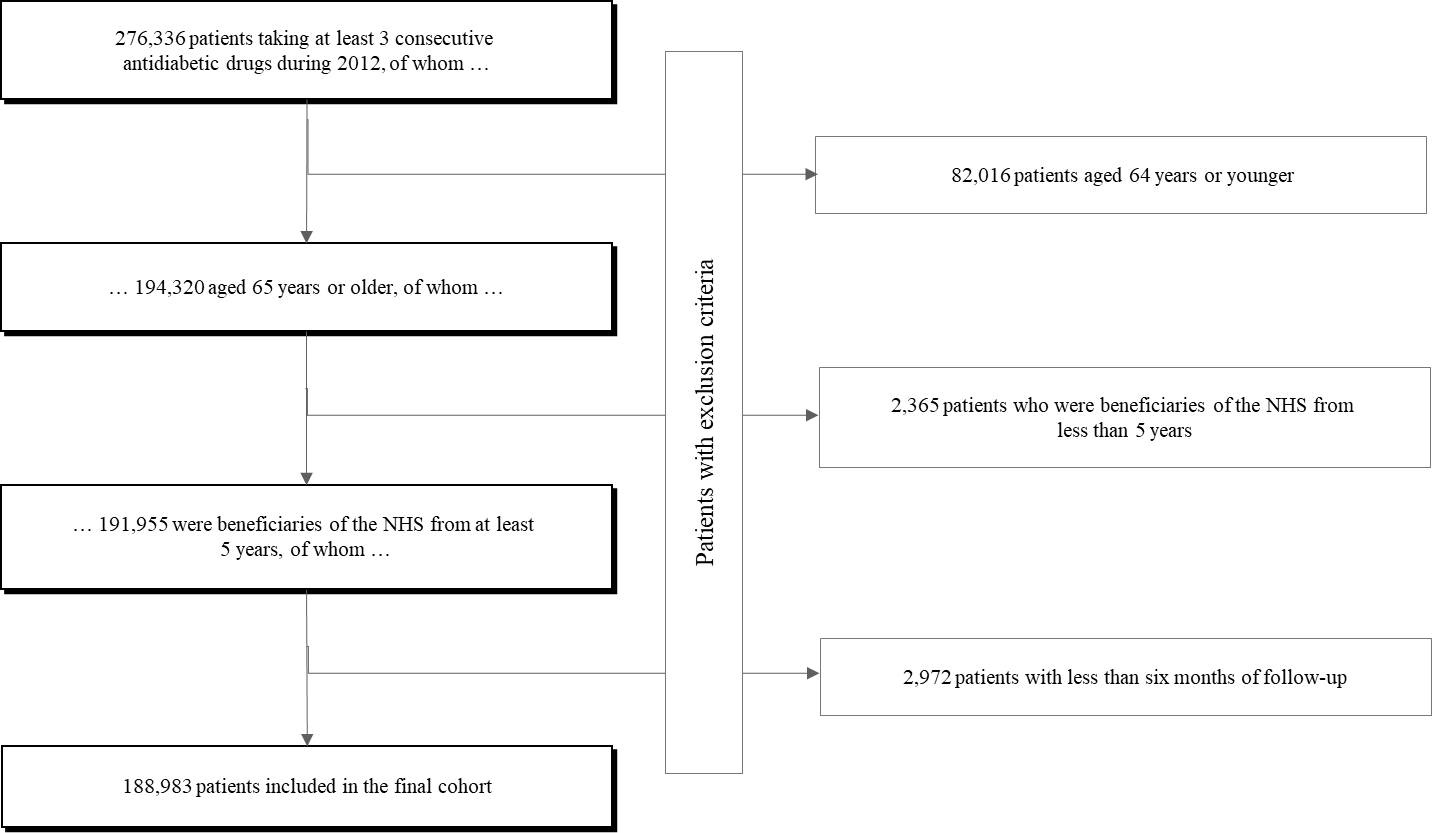


**Supplementary Figure S2 –** Influence of a confounder on the relationship between adherence to antidiabetic drugs (exposure) and all-cause death in the different clinical categories as determined by MCS

The graph indicates the RR_CO_ or RR_CE_ combinations (i.e. the confounder outcome and the confounder-exposure associations, respectively) that would be required to move the observed protective effects of high adherence towards the null in the different clinical categories. The confounder’s prevalence in the study population was set at 30%.
